# The Phytochemical Rhein Mediates M^6^A-Independent Suppression of Adipocyte Differentiation

**DOI:** 10.3389/fnut.2021.756803

**Published:** 2021-11-01

**Authors:** Linyuan Huang, Jun Zhang, Xinyun Zhu, Xue Mi, Qiujie Li, Jing Gao, Jianheng Zhou, Jun Zhou, Xiao-Min Liu

**Affiliations:** ^1^School of Life Science and Technology, China Pharmaceutical University, Nanjing, China; ^2^State Key Laboratory of Natural Medicines, China Pharmaceutical University, Nanjing, China

**Keywords:** rhein, *N*^6^-methyladenosine, adipocyte, differentiation, mitotic clonal expansion, REEP3

## Abstract

Adipogenesis is mediated by the complex gene expression networks involving the posttranscriptional modifications. The natural compound rhein has been linked to the regulation of adipogenesis, but the underlying regulatory mechanisms remain elusive. Herein, we systematically analyzed the effects of rhein on adipogenesis at both the transcriptional and posttranscriptional levels. Rhein remarkably suppresses adipogenesis in the stage-specific and dose-dependent manners. Rhein has been identified to inhibit fat mass and obesity-associated (FTO) demethylase activity. Surprisingly, side-by-side comparison analysis revealed that the rhein treatment and *Fto* knockdown triggered the differential gene regulatory patterns, resulting in impaired adipocyte formation. Specifically, rhein treatment mildly altered the transcriptome with hundreds of genes dysregulated. *N*^6^-methyladenosine (m^6^A) methylome profile showed that, although the supply of rhein induced increased m^6^A levels on a small subset of messenger RNAs (mRNAs), few of them showed dramatic transcriptional response to this compound. Moreover, the specific rhein-responsive mRNAs, which are linked to mitotic pathway, are barely methylated or contain m^6^A peaks without dramatic response to rhein, suggesting separate regulation of global m^6^A pattern and adipogenesis mediated by rhein. Further identification of m^6^A-independent pathways revealed a positive regulator, receptor expressing-enhancing protein 3 (REEP3), in guidance of adipogenesis. Hence, this study provides the mechanistic view of the cellular actions of rhein in the modulation of adipogenesis and identifies a potential novel target for obesity therapeutic research.

## Introduction

Obesity, characterized by excessive accumulation of adipose, is becoming a global health problem in the society of today. Adipose, also known as a tissue for storing lipids, is now defined as a dynamic organ involved in many critical physiological processes such as energy homeostasis, hormone secretion, and insulin sensitivity (1–4). Adipose tissue mainly consists of adipocytes that primarily develop from preadipocytes in a highly coordinated progression called adipogenesis (5). The adipocyte differentiation is typically triggered by the adipogenic induction cocktail containing 3-isobutyl-1-methylxanthine (IBMX), dexamethasone, and insulin (MDI) and divided into three consecutive stages: growth arrest, mitotic clonal expansion (MCE), and terminal differentiation (6, 7). Previous studies have indicated that MCE is required to induce adipogenesis of several cell models, among which 3T3-L1 is the most widely used. Blocking the MCE progression severely obstacles the marker gene expression in cell cycle progression and adipogenic activation leading to attenuated adipocyte differentiation (8).

Rhein is a bioactive molecule isolated from rhizomes of medicinal plants such as *Rheum L*. As a substance in the anthraquinone group, rhein is commonly used as a cathartic (9). It not only sensitizes the carcinoma cells to chemotherapy or radiotherapy (10–13), but also exhibits excellent anti-inflammatory activities (14) and plays significant roles in the protection of the myocardial cells from hypoxia-induced injury (15, 16). Additionally, the phytochemical rhein is involved in the regulation of the adipogenesis process in the cultured cells (17, 18). It has been shown to inhibit the *in-vitro* differentiation of 3T3-L1 preadipocytes through downregulating adipogenesis marker genes such as *Ppar*γ and *C/ebp*α (17). However, the mechanistic action of this compound on the cellular gene regulatory pathways remains to be systematically investigated.

Recently, it has been reported that rhein is capable of binding to a AlkB protein, fat mass and obesity-associated (FTO) protein gene, and inhibiting its enzymatic activity (19). A genome-wide association study revealed that the single-nucleotide polymorphisms in FTO region are correlated with body mass in multiple populations (20, 21). Intriguingly, FTO is the first demethylase identified to remove the *N*^6^-methyladenosine (m^6^A) on messenger RNA (mRNA) (22, 23). A number of studies have indicated that FTO plays certain role in the regulation of adipocyte generation *via* targeting m^6^A-containing regulatory mRNAs (24–31). For example, FTO could mediate exonic splicing of adipogenic regulatory factor RUNX1T1 through adjusting m^6^A abundance around splice sites and, thus, regulating differentiation (26). Whether rhein could rearrange global m^6^A methylome landscape, and whether FTO-mediated demethylation participates in rhein-interfered adipocyte differentiation remain elusive

In this study, we conducted a systematic investigation of rhein actions on adipogenesis by taking advantage of the transcriptional and posttranscriptional approaches. We showed that stage-specific supply of rhein dramatically attenuated adipocyte differentiation. Comparative analysis of rhein treatment and *Fto* knockdown revealed that rhein-specific responsive mRNAs are associated with the mitotic pathway. Further combinational data analysis indicated that rhein regulated m^6^A methylome rearrangement and adipogenesis in an independent manner. Finally, we identified a m^6^A-independent regulator, which is positively involved in the modulation of adipocyte differentiation.

## Materials and Methods

### Cell Culture, Adipocyte Differentiation, and Rhein Treatment

3T3-L1 preadipocytes were cultured in the Dulbecco's Modified Eagle Medium (DMEM) (Gibco 11995073, Carlsbad, CA) with 10% newborn calf serum (Gibco 26010074, Carlsbad, CA) and 1% penicillin-streptomycin (PS) (Gibco 15140163, Carlsbad, CA). About 2 days after confluency, cells were fed with MDI induction media containing DMEM, 10% fetal bovine serum (FBS) (Gibco 10099141C, Carlsbad, CA), 1% PS, 0.5 mM IBMX (Sigma I7018, St. Louis, Missouri), 1 μM dexamethasone (Sigma D4902, St. Louis, Missouri), and 10 μg/ml insulin (Sigma I0516, St. Louis, Missouri) for 3 days. Then, cells were replenished with maintenance media supplied with 10% FBS, 1% PS, and 10 μg/ml insulin every 2–3 days until the fully differentiated adipocyte-like cells were obtained. 20 μM rhein treatment was also carried out in this study.

### Oil Red O Staining

Mature adipocytes were rinsed with phosphate-buffered saline (PBS) and fixed with 10% formalin at room temperature for 1 h. The fixed cells were then rinsed with PBS and equilibrated with 60% isopropanol diluted in double-distilled water (ddH_2_O). After staining with the ORO working solution at room temperature for 10 min, cells were washed with ddH_2_O until the supernatant was clear. The stained cells were photographed by using a microscope (Nikon, Tokyo). ImageJ software was applied to analyze the relative lipid accumulation.

### Short Hairpin RNA Knockdown Cell Lines Construction

Lentiviral shRNA templates were cloned into shRNA expression vector termed as the DECIPHER pRSI9-U6-(sh)-UbiC-TagRFP-2A-Puro (Cellecta, California, USA). shRNA lentivirus was produced by using Lenti-X 293T cells and harvested 48 h after transfection. 3T3-L1 cell lines were infected by the filtered viral media for 48 h with 10 μg/ml polybrene (Sigma, St. Louis, Missouri). The complete growth media containing 2 μg/ml puromycin were used to select cells. Lentiviral shRNA targeting sequences are listed below:

shScramble: 5′-AACAGTCGCGTTTGCGACTGG-3′
sh*Fto*-1: 5′-GCTGAGGCAGTTCTGGTTTCA-3′
sh*Fto*-4: 5′-GACATCGAGACACCAGGATTA-3′
sh*Reep3*-1: 5′-CCCAGCAATGACTAACATCTT-3′
sh*Reep3*-2: 5′-CTATGAGACAATGGTGAATTT-3′

### Quantitative Real-Time PCR Analysis

Total RNA was extracted by using the TRIzol reagent (Invitrogen 15596018, Waltham, Massachusetts) and the first strand complementary DNA (cDNA) was synthesized *via* the HiScript III 1st Strand cDNA Synthesis Kit (Vazyme R312-02, Nanjing, Jiangsu). qPCR analysis was carried out by using the ChamQ SYBR Green qPCR Master Mix (Vazyme A331-02, Nanjing, Jiangsu) by using the QuantStudio^TM^ 3 Real-Time PCR Instrument (Applied Biosystems, Waltham, Massachusetts). Primers used for amplifying target genes were presented in Supplementary Table 1.

### Western Blot Analysis

Cells were rinsed with PBS and lysed in sodium dodecyl sulfate-polyacrylamide gel electrophoresis (SDS-PAGE) sample buffer (62.5 mM Tris-HCl, pH 6.8, 2% SDS, 25% glycerol, 5% β-mercaptoethanol, and 0.01% bromophenol blue) followed by heating at 100°C for 8 min. Then, samples were loaded on SDS-PAGE gels and separated by electrophoresis before being transferred to polyvinylidene difluoride (PVDF) membranes (Millipore IPVH00010, Burlington, Massachusetts). The membranes were first blocked by 5% non-fat milk diluted in 0.1% Tris buffered saline, 0.1% Tween (TBST) (1 × TBS, 0.1% Tween-20) for 1 h at room temperature followed by incubating with desired primary antibodies diluting in 5% non-fat milk at 4°C overnight. After that, membranes were rinsed three times with 0.1% TBST and incubated with corresponding horseradish peroxidase (HRP)-conjugated secondary antibodies for 1 h at room temperature. The primary antibody signals were visualized after reaction with enhanced chemiluminescence (Vazyme E412-02, Nanjing, Jiangsu). The primary antibodies used in western blot analysis were presented below: anti-β-actin (Proteintech 60008-1-Ig, Rosemont, Illinois), antiperoxisome proliferator-activated receptor γ (PPARγ) (Proteintech 16643-1-AP, Rosemont, Illinois), antifatty acid-binding protein 4 (FABP4) (Santa Cruz sc-271529, Santa Cruz, CA), anti-FTO (Abcam ab92821, Cambridge, England), anti-ALKBH5 (Proteintech 16837-1-AP, Rosemont, Illinois), anticyclin A (Proteintech 18202-1-AP, Rosemont, Illinois), and anticyclin B1 (Proteintech 55004-1-AP, Rosemont, Illinois).

### *N^6^*-Methyladenosine Dot Blot

Total RNA was extracted by using the TRIzol Reagent first and mRNA was isolated by using the Oligo d(T)25 Magnetic Beads (NEB S1419S, Ipswich, Massachusetts). A 2-fold serial dilution starting from 500 ng of mRNA was spotted on a nylon membrane and crosslinked under 254 nm UV light (0.12 J/cm^2^). Subsequently, the membrane was blocked by 5% non-fat milk diluted in 0.1% PBST (1 × PBS, 0.1% Tween-20) for 1 h at room temperature followed by incubating with anti-m^6^A antibody (Abcam ab151230, Cambridge, England) 1:1000 dilution at 4°C overnight. After that, the membrane was rinsed three times with 0.1% PBST and incubated with HRP-conjugated antirabbit immunoglobulin G (IgG) for 1 h at room temperature. The m^6^A blotting signal was visualized after reaction with enhanced chemiluminescence.

### Ribonucleic Acid Sequencing and MeRIP-Seq

For RNA-seq and m^6^A RNA immunoprecipitation followed by high-throughput sequencing (MeRIP-seq), cells treated with rhein were collected at day 0 (preadipocytes), day 3 (MCE), and day 10 (differentiation), respectively. Total RNA was extracted by using the TRIzol Reagent followed by chemical fragmentation by using the freshly prepared fragmentation buffer as previously described approaches (32–34). A portion of 5 μg untreated fragmented RNA was served as input control for RNA-seq and 600 μg fragmented RNA was subjected to MeRIP-seq. For MeRIP-seq, the fragmented RNA was incubated with 12 μg anti-m^6^A antibody (Abcam ab151230) in 1 × immunoprecipitation (IP) buffer [10 mM Tris-HCl, pH 7.4, 150 mM sodium chloride (NaCl), 0.1% NP-40, and 40 U/μl RNase inhibitor] with continuous rotation for 2 h at 4°C. In the meanwhile, 15 μl protein A and 15 μl protein G mixed beads were blocked in 1 × IP buffer supplemented with bovine serum albumin (BSA) (0.5 mg/ml). After that, the mixtures were incubated with the prior blocked beads on a rotating wheel for another 2 h at 4°C. Following that, the reaction mixtures were spinned down and sediments were eluted twice in 100 μl of elution buffer with 6.7 mM m^6^A 5′-monophosphate sodium salt (Santa Cruz sc-215524, Santa Cruz, CA) in 1 × IP buffer followed by ethanol precipitation. The input fragmented RNA and precipitated m^6^A elute were used for cDNA library construction and deep sequencing described below.

### Complementary DNA Library Construction

The desired m^6^A-enriched RNA elute and input control were used to construct cDNA library in parallel as described in our previous studies (35, 36). Briefly, samples were first dephosphorylated and 40–60 nt region was selected according to 15% polyacrylamide Tris-borate-EDTA (TBE)-urea gel separation. Desired RNA species were excised and soaked overnight in gel elution buffer [300 mM sodium acetate (NaOAc), pH 5.5, 1 mM ethylenediaminetetraacetic acid (EDTA), and 0.1 U/ml SUPERase_In) followed by ethanol precipitation. Then, the purified RNA was processed for poly-(A) tailing reaction and reverse transcription afterward. Eventually, PCR amplification was proceeded by using the Phusion High-Fidelity DNA Polymerase (NEB M0530L, Ipswich, Massachusetts). The quantification of PCR products was evaluated on the Agilent 2100 Bioanalyzer System (Santa Clara, CA) before being subjected to cluster generation followed by deep sequencing on an Illumina Novaseq platform (San Diego, CA).

### Ribonucleic Acid Sequencing Data Processing

The Trim Galore (v0.6.5) was initially used to trim the low-quality sequences and retain paired reads under—length 100—paired parameters. The paired-end reads were aligned to the mouse genome (GRCm38) with the HISAT2 aligner (v2.1.0) with default parameters and *Ensembl* genes (GRCm38.102) transcriptome annotation (37). Then, gene expression was quantified by using the StringTie (v2.1.4) (38) and differentially expressed genes were identified by using the DESeq2 (v1.30.1) (39). The fold change > 2 or <0.5 and *p* < 0.05 genes were considered as differentially expressed genes. The transcripts per kilobase of exon model per million mapped reads (TPM) was used to produce heatmap.

### MeRIP-Seq Data Processing

Quality control of MeRIP-seq data was performed by using the FastQC (v.0.11.9). Low-quality bases and adaptor contamination were removed by the Trim Galore. Remaining reads were aligned to mouse genome version mm10 with the HISAT2 aligner with default parameters. The mapped reads were filtered with the Picard (v2.23.5) to remove PCR duplicates and the SAMtools (v1.9) to remove the reads with mapping quality below 30. m^6^A peaks were identified by using the exomePeak2R package with default parameters. Peaks were annotated by the R package ChIPseeker (v.1.26) (40). The distribution of m^6^A peaks was plotted by the R package Guitar (v.2.6.0). Motif finding was performed by using the HOMER (v.4.11) findMotifs.pl with parameter (-rna -p 10 -len 5, 6, 7, 8 -S 10). The final bam files were transformed to bigwig format by using the deepTools (v3.5.0) with parameter [–normalizeUsing Bins Per Million (BPM)]. The Integrative Genomics Viewer (IGV) (v2.94) was used to visualize the aligned data.

### Enrichment Analysis

The Gene Ontology (GO) and the Kyoto Encyclopedia of Genes and Genomes (KEGG) pathway analysis were done on the DAVID (https://david.ncifcrf.gov) with default parameters. The gene set enrichment analysis (GSEA) software (v4.1.0) was used for the analysis of gene expression data with (c5.go.bp.v7.3.symbols.gmt) gene set with default settings (41).

### Statistical Analysis

Statistical analysis was carried out by using the GraphPad Prism. Unless otherwise specified, all the values were conducted as the mean ± SEM. Differential analysis was calculated by the Student's *t*-test. The data were considered as statistically significant when ^*^p < 0.05, ^**^p < 0.01, or ^***^p < 0.001.

### Data Availability

The raw and processed high-throughput sequencing data in this study are publicly available in the Gene Expression Omnibus (GEO) (reference numbers GSE171785 and GSE173702).

## Results

### Stage-Specific Treatment of Rhein Attenuates Adipocyte Formation in a Dose-Dependent Manner

**Figure 1:**
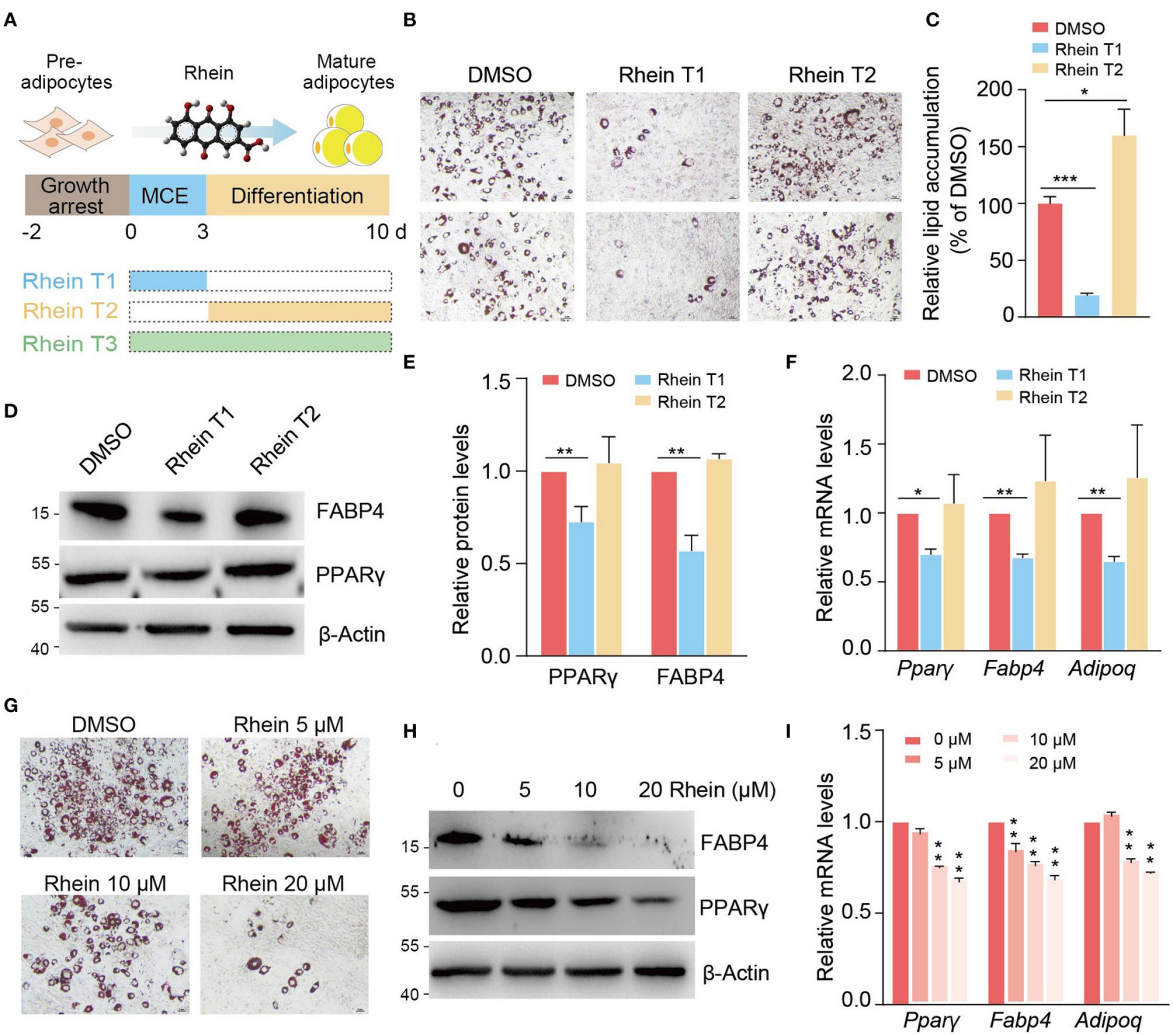
Rhein suppresses adipogenesis at specific stage in a dose-dependent manner. **(A)** Schematic representation of rhein treatment at the different stages during adipocyte differentiation. **(B)** The adipogenic phenotypes of 3T3-L1 cells treated with rhein at the indicated stages. Cells cultured with dimethyl sulfoxide (DMSO) were served as a negative control. Lipid accumulation was assessed by using Oil Red O staining; scale bars, 5 μm. **(C)** Quantification of lipid accumulation in **(B)**. Error bars, mean ± SEM; *n* = 8 biological replicates; *p < 0.05, ***p < 0.001. **(D)** Western blotting of the adipogenic markers. Cells were treated with DMSO or rhein before being collected for analysis. For all the western blots in this study, molecular weight markers (kDa) are indicated on the left side. **(E)** Quantification of the protein levels shown in **(D)**. Error bars, mean ± SEM; *n* = 3 biological replicates; **p < 0.01. **(F)** Relative messenger RNA (mRNA) levels of adipogenesis markers upon DMSO or rhein treatment. Error bars, mean ± SEM; *n* = 4 biological replicates; *p < 0.05, **p < 0.01. **(G)** The adipogenic phenotypes of 3T3-L1 cells treated with rhein at indicated concentrations. Cells cultured with DMSO were served as a negative control. Lipid accumulation was assessed by using Oil Red O staining; scale bars, 5 μm. **(H)** Western blotting of adipogenic marker proteins in cells cultured with MDI medium in the presence of various concentrations of rhein at the mitotic clonal expansion (MCE) stage. **(I)** Effect of rhein treatment with various concentrations on mRNA levels of adipogenesis markers. Error bars, mean ± SEM; *n* = 3 biological replicates; **p < 0.01.

Rhein has been suggested to regulate adipogenesis and mice body weight in the previous studies. To systematically evaluate the effect of rhein on adipocyte differentiation, we induced adipogenesis of growth-arrested 3T3-L1 preadipocytes with the MDI medium in the presence or absence of rhein. Rhein treatment was carried out at the different stages including the MCE stage (rhein T1), the differentiation stage (rhein T2), and continuous treatment at both the stages (rhein T3) (Figure 1A). Remarkably, as shown in ORO staining results, rhein treatment at the early stage effectively inhibited the mature adipocytes formation, as lipid droplets accumulation in the mature adipocytes reduced 80% in the rhein T1 group compared to the dimethyl sulfoxide (DMSO) control, whereas supplement of the phytochemical at the later stage (rhein T2 group) could not attenuate the subsequent adipocytes formation (Figures 1B,C). Consistent with this, the expressions of the master regulator PPARγ in adipogenesis were significantly decreased in the rhein T1 group compared to the rhein T2 group. Spontaneously, three major downstream target genes of *Ppar*γ, *Fabp4*, and *Adiponectin* (*Adipoq*) were also responsively downregulated (Figures 1D–F). To the best of our knowledge, the prolonged treatment of rhein at both the MCE stage and the differentiation stage had negligible influence on the adipogenic progression (Supplementary Figures 1A–C), indicating that the MCE stage is the crucial period for rhein to exert its detrimental effects on adipocytes development. Therefore, we conducted the rhein treatment at the MCE stage in the subsequent experiments.

Next, we investigated whether the different doses of rhein could similarly influence the progression of preadipocyte differentiation. As shown in Figures 1G–I, the newly formed adipocytes coupled with the mRNA and protein expressions of three marker genes (*Ppar*γ, *Fabp*, and *Adipoq*) were gradually decreased with increasing concentration of rhein. All the results above suggest that rhein modulates the adipogenesis process in stage-specific and dose-dependent manners.

### Rhein Triggers Fat Mass and Obesity-Associated-Dependent and Fat Mass and Obesity-Associated-Independent Global Transcriptome Changes

Rhein has been identified to inhibit FTO demethylase activity. To investigate whether rhein suppresses the adipogenic process through targeting FTO, we initially verified the functional role of FTO in adipogenesis. As examined in ORO staining, knockdown of *Fto* resulted in a substantial decrease in the accumulation of the lipid droplets of the mature adipocytes (Supplementary Figure 2A). Meanwhile, the RNA levels of adipogenesis marker genes *Fabp4* and *Adipoq* were significantly downregulated upon FTO deficiency (Supplementary Figures 2B,C), confirming the positive functional role of FTO in adipocyte formation.

**Figure 2:**
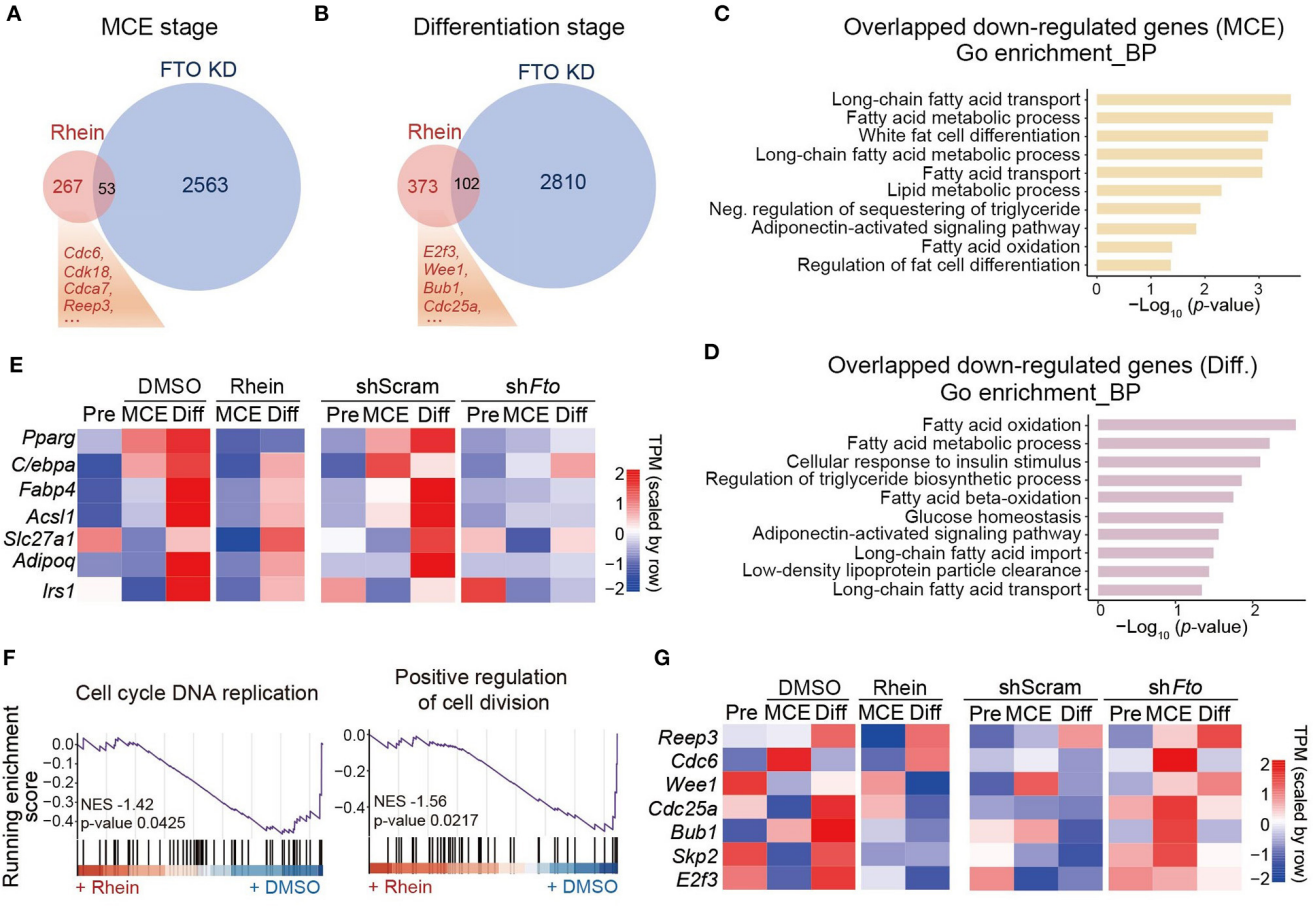
Comparative transcriptome analysis of effects of rhein treatment and fat mass and obesity-associated (*Fto*) depletion on adipogenesis. **(A)** Venn diagram showing the overlap of downregulated genes in cells lacking *Fto* or treated with rhein at the MCE stage. **(B)** Venn diagram showing the overlap of downregulated genes in cells lacking *Fto* or treated with rhein at the differentiation stage. **(C,D)** The Gene Ontology (GO) analysis of common downregulated genes in rhein-treated and sh*Fto* cells collected at the MCE stage or the differentiation stage. **(E)** Heatmap showing expression of genes responsible for adipocyte formation in rhein-treated cells and *Fto* knockdown cells at the distinct stages. **(F)** The gene set enrichment analysis plots for rhein-regulated genes, as determined by RNA-seq. **(G)** Heatmap showing expression of genes responsible for mitosis in rhein-treated and sh*Fto* cells at the distinct stages.

Next, we sought to compare the global gene expression profiles in rhein-treated cells with those in *Fto*-knockdown cells during the process of adipocyte differentiation. At the MCE and the differentiation stages, knockdown of *Fto* led to over 4,000 differentially expressed genes (DEGs) compared to the control cells (shScram) (Supplementary Figures 3A,B). However, only hundreds of genes changed expression in response to rhein treatment (Supplementary Figures 3C,D). There were 53 and 102 genes commonly downregulated in both the groups at the MCE stage and differentiation stage, respectively (Figures 2A,B). As expected, the GO analysis revealed that these overlapped genes were involved in the fat cell differentiation process, fatty acid metabolic process, and PPARγ signaling pathway (Figures 2C,D and Supplementary Figures 4A,B). Consistent with our previous results, the primary adipogenic marker genes and their regulatory genes, *Fabp4, Adipoq, C/ebp*β, and *Ppar*γ, were included in the overlap group (Figure 2E). A close inspection of the MCE stage revealed that only 16.5% (53 out of 320) of genes downregulated upon rhein also showed reduced expression in response to FTO deficiency (Figure 2A). The similar pattern was also observed at the differentiation stage. Only 21.5% of genes in the rhein-downregulated group bore decreased expression upon *Fto* knockdown (Figure 2B), suggesting that rhein might primarily target FTO-independent regulatory pathway to interfere adipocyte differentiation.

We, therefore, focused on the genes that are specifically downregulated in the rhein-treated group. As overlapped genes, rhein-specific downregulated genes were associated with positive regulation of fat cell differentiation by using the GSEA (Supplementary Figures 4C,D). In addition, the GSEA analysis also revealed that these genes participated in the cell cycle and cell division pathway (Figure 2F), suggesting that rhein treatment might disturb mitotic progression. We further took a close look at the mitotic-related genes. In contrast to the adipogenic genes, they were obviously downregulated upon rhein treatment rather than FTO deficiency, especially at differentiation stage (Figure 2G). These observations collectively indicate that besides minimal FTO-dependent regulatory genes, rhein could limit the expressions of FTO-independent mitotic genes as well to attenuate the adipocyte formation.

### Rhein Retards the Mitotic Clonal Expansion Progression During Adipogenic Differentiation

**Figure 3:**
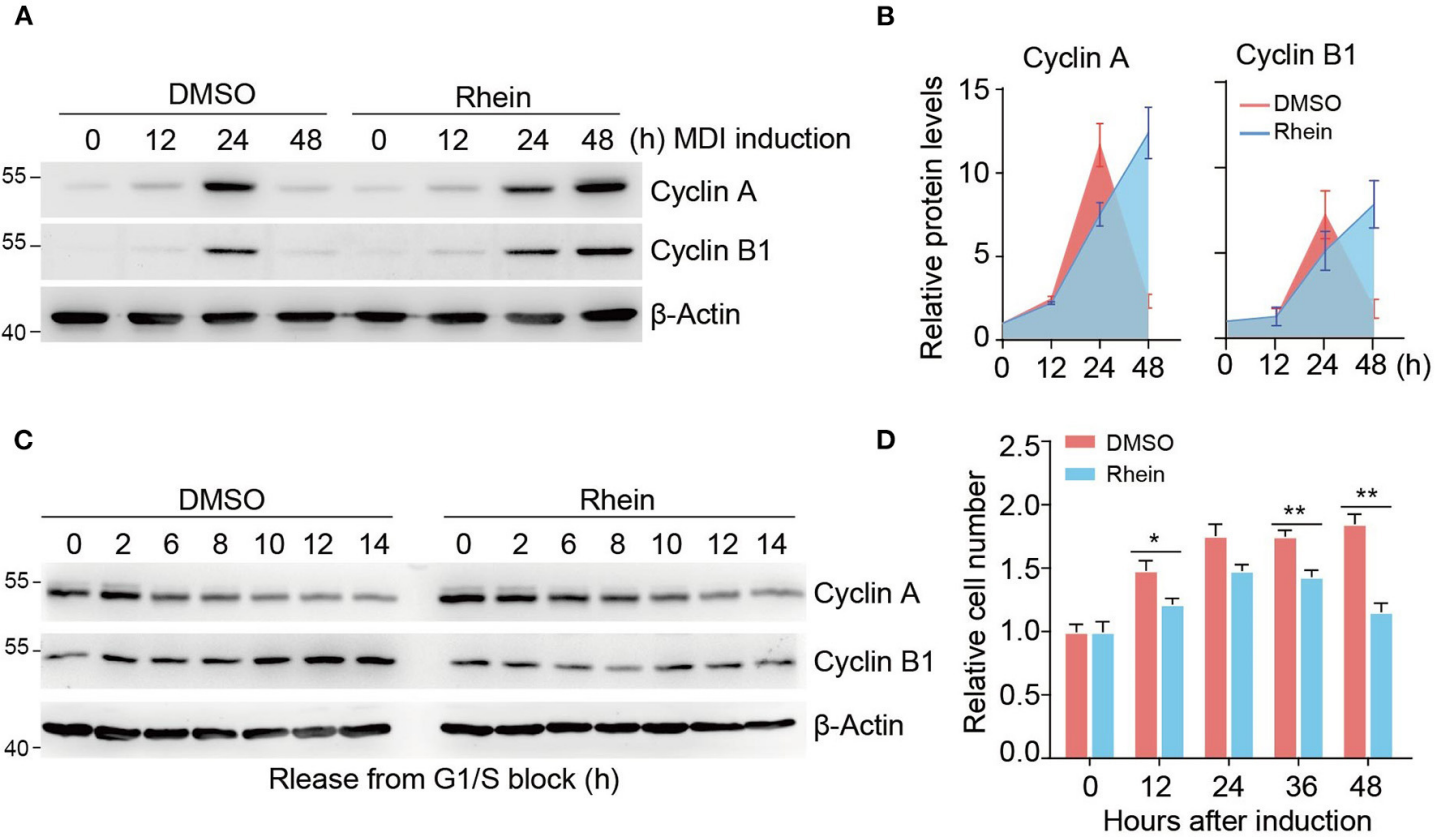
Rhein impairs the MCE progression during differentiation. **(A)** Immunoblotting of cell-cycle markers during MCE. Cells cultured in the MDI medium with or without rhein were collected at indicated time points for analysis. **(B)** Quantification of protein levels shown in **(B)**. Error bars, mean ± SEM; *n* = 3 biological replicates. **(C)** Immunoblotting of cell-cycle markers in cells released from G1/S boundary. Cells were arrested by using double thymidine block, released into fresh medium, and harvested at the indicated time points. **(D)** The effect of rhein on cell proliferation during MCE. Growth-arrested preadipocytes were induced by MDI medium and cell number was determined at indicated time points. Error bars, mean ± SEM; *n* = 6 biological replicates; *p < 0.05, **p < 0.01.

We have shown that rhein treatment results in remarkable reduced expressions of genes involved in the mitotic pathway. To confirm whether rhein plays a role in the regulation of MCE progression, we detected two typical mitotic markers, cyclin A and cyclin B1, during MDI-induced adipogenic formation. As shown in Figure 3A, both cyclin A and cyclin B1 proteins accumulated at highest levels at 24 h after the addition of MDI medium (Figure 3B). The presence of rhein delayed the maximal induction of cyclin proteins with their expression peaks at 48 h, suggesting that rhein treatment retards the progression of the cell cycle.

To further validate the functional role of rhein in the regulation of mitotic progression, we initially synchronized 3T3-L1 cells at G1/S boundary by using a double thymidine block. After release by addition of fresh medium, cells went through the cell cycle synchronously from the G1/S boundary to mitosis until the subsequent G1 phase. In the absence of rhein, levels of both cyclin expression fluctuated during the cell cycle. Specifically, the cyclin A protein was present at low levels at 6 h, while cyclin B1 abundance increased gradually (Figure 3C). Intriguingly, rhein treatment at the MCE stage remarkably prolonged cyclin A degradation and retarded cyclin B1 expression, highlighting the negative function of rhein in the regulation of cell cycle progression. Further supporting this notion, rhein-treated 3T3-L1 cells proliferated more slowly compared to the control cells (Figure 3D). Collectively, these results provide strong evidence indicating that rhein retards the MCE progression to suppress adipogenic differentiation.

### Rhein Reshapes the Landscape of *N^6^*-Methyladenosine Methylome

**Figure 4:**
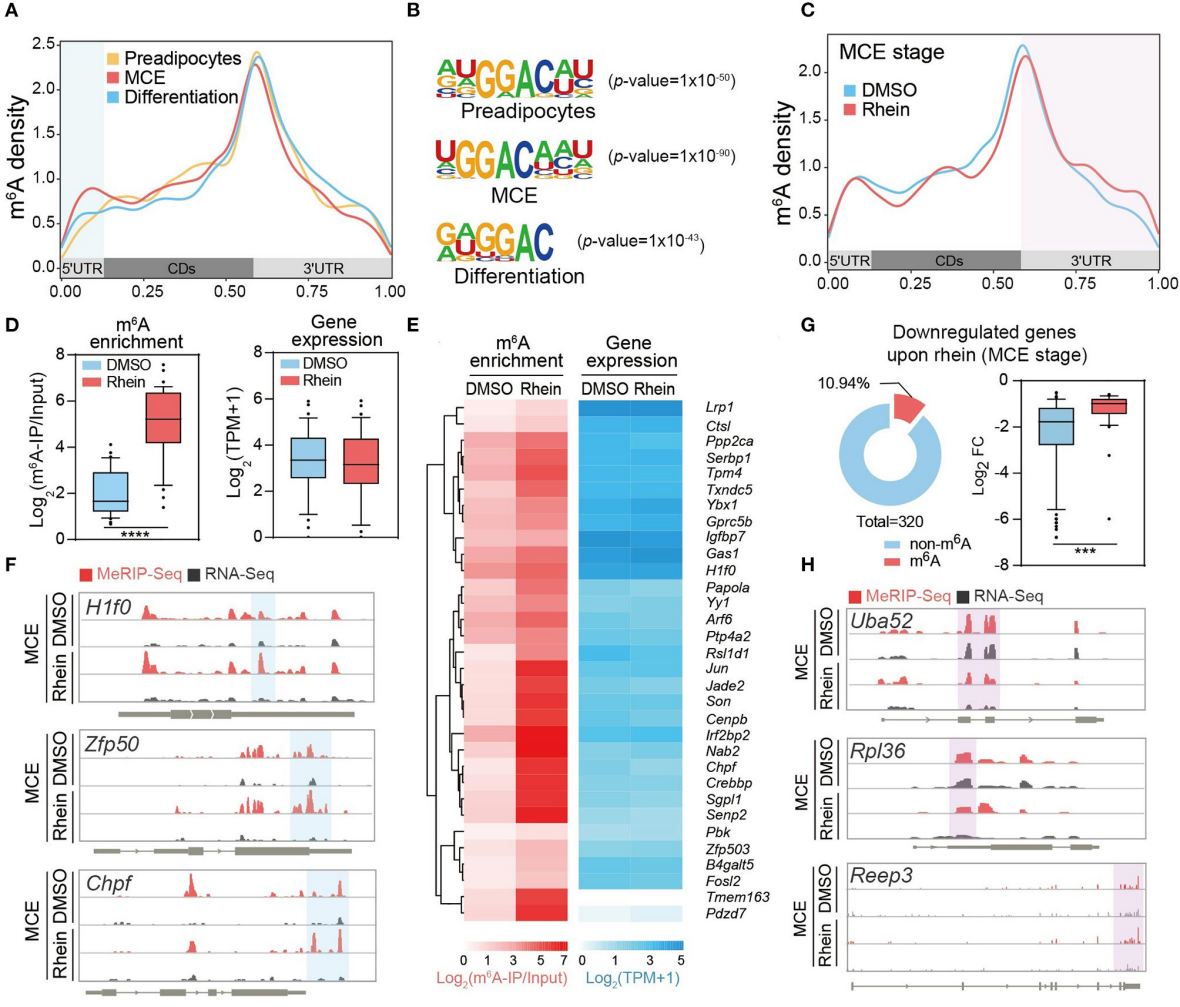
Rhein rearranges the landscape of *N*^6^-methyladenosine (m^6^A) methylome. **(A)** Metagene profiles of m^6^A distribution across the transcriptome of cells at the preadipocytes, the MCE stage, or the differentiation stage, respectively. **(B)** Sequence logo representing the consensus motif relative to m^6^A at the various stages in response to rhein or DMSO, respectively. **(C)** Metagene profiles of m^6^A distribution across the transcriptome of cells treated with DMSO or rhein at the MCE stage. **(D)** Box plot showing the fold changes of m^6^A enrichment and RNA levels in genes with elevated m^6^A levels (fold change > 2) upon rhein treatment. **(E)** Heatmap showing m^6^A enrichment and expression levels of genes with increased m^6^A at the MCE stage upon rhein treatment. **(F)** Examples of rhein-regulated m^6^A peaks. In an Integrative Genomics Viewer (IGV) format, normalized m^6^A-IP and input control coverage are shown in red and gray, respectively. Exons are represented by thick black boxes; 5′ and 3′ untranslated regions (UTRs) are represented by thin black boxes; introns are represented by thin lines. **(G)** Ring chart showing the ratio of m^6^A-modified transcripts in genes downregulated by rhein at the MCE stage. Box plot showing the fold changes of RNA levels in genes (fold change > 2) downregulated by rhein treatment. **(H)** Examples of m^6^A-independent transcriptional downregulation. In an IGV format, normalized m^6^A-IP and input control coverage are shown in red and gray, respectively. Exons are represented by thick black boxes; 5′ and 3′ UTRs that are represented by thin black boxes; introns are represented by thin lines. ***p < 0.001, ****p < 0.0001.

We next characterized the m^6^A dynamics during the adipogenesis process. As shown in dot blot assay (Supplementary Figure 5A), m^6^A levels underwent dynamic increase during adipogenic differentiation, which is consistent with the previous study (26). Global m^6^A methylome analysis revealed typical enrichment of m^6^A in 3′ untranslated region (UTR) and near stop codon along the transcripts at the three different stages during adipogenesis (Figure 4A). Besides, *de-novo* motif analysis by using program Multiple Em for Motif Elicitation (MEME) identified the consensus sequence “GGAC” (Figure 4B). Surprisingly, a close inspection of methylomes showed that m^6^A read density increased dramatically at 5′ UTR, but not 3′ UTR or coding regions (CDs) of mRNAs during adipogenic differentiation (Figure 4A). Consistent with this, the percentage of m^6^A peaks within 5′ UTR was relatively high at the later two stages compared to the growth arrest stage (Supplementary Figure 5B). Altogether, these results suggest that m^6^A plays crucial roles in the regulation of adipogenic formation.

We tend to investigate how rhein regulates the global m^6^A methylome. As a potential FTO inhibitor, rhein treatment triggered an increased abundance of m^6^A, but not dysregulated expressions of m^6^A writer and eraser proteins (Supplementary Figures 6A,B), suggesting that rhein mainly interferes with the catalytic activity rather than protein abundance of FTO. We focused on the comparative m^6^A methylome in the presence and absence of rhein at the MCE stage, as rhein treatment substantially suppresses the mitotic progression. Unexpectedly, global m^6^A peak analysis revealed a specific increase of m^6^A read density at 3′ UTR after rhein treatment (Figure 4C), suggesting that rhein and differentiation signal possibly perturb the global m^6^A methylome in a distinct manner.

### Rhein Separately Modulates RNA Methylation and Adipogenesis

Next, we sought to assess whether rhein modulates gene expression in an m^6^A-dependent manner. As rhein may also induce the upregulation of m^6^A levels at the other regions of individual transcripts through acting on FTO, we analyzed all the mRNAs with significantly increased m^6^A at any regions upon rhein. These transcripts showed similar abundance before and after rhein treatment (Figure 4D). Individually, the majority of m^6^A-containing transcripts were insensitive to be dysregulated upon rhein such as *H1f0, Zfp50*, and *Chpf* (Figures 4E,F). On the other hand, we deeply analyzed the 320 MCE stage-specific rhein-downregulated transcripts, which are involved in the mitotic pathway. Only 10.94% of these mRNAs contained m^6^A peaks (Figure 4G), while the majority of transcripts did not bear m^6^A peaks as exemplified by *Uba52, Rpl36*, and receptor expressing enhancing protein 3 (*Reep3)* (Figure 4H). Furthermore, m^6^A-bearing transcripts were much less sensitive to rhein compared to non-m^6^A transcripts (Figure 4G). Collectively, the available evidence suggests that rhein modulates global RNA methylation landscape and adipogenic differentiation in a separate manner.

### Receptor Expressing-Enhancing Protein 3 Is Required for Adipogenic Differentiation

**Figure 5:**
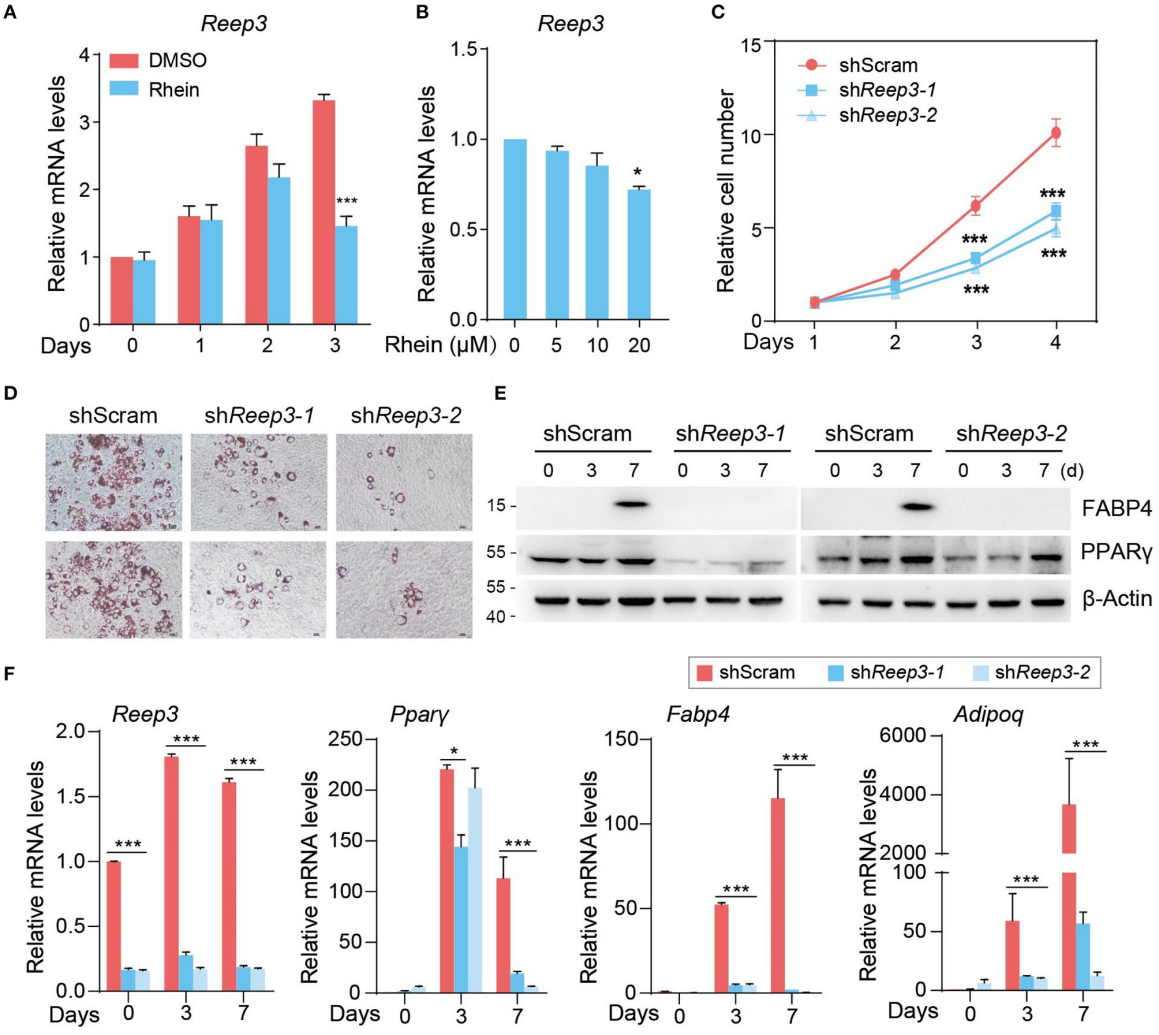
Receptor expressing-enhancing protein 3 (REEP3) is essential for efficient adipocyte differentiation. **(A)** Relative mRNA levels of *Reep3* under DMSO or rhein treatment during the MCE stage. Error bars, mean ± SEM; *n* = 4 biological replicates; ***p < 0.001. **(B)** Effect of rhein treatments with various concentrations on mRNA levels of *Reep3*. Error bars, mean ± SEM; *n* = 4 biological replicates; *p < 0.05. **(C)** The proliferation rate of shScram and sh*Reep3*3 cells. Error bars, mean ± SEM; *n* = 3 biological replicates; ***p < 0.01. **(D)** The adipogenic phenotypes of sh*Reep3* and shScram cells. Lipid accumulation was assessed by using Oil Red O staining; scale bars, 5 μm. **(E)** Western blotting of fatty acid-binding protein 4 (FABP4) and peroxisome proliferator-activated receptor γ (PPARγ) in sh*Reep3* and shScram cells. **(F)** Relative mRNA levels of *Reep3, Ppar*γ, *Fabp4*, and adiponectin (*Adipoq*) in knockdown (sh*Reep3-1 and* sh*Reep3-2*) and control (shScram) cells. Error bars, mean ± SEM; *n* = 4 biological replicates; *p < 0.05, ***p < 0.001.

We focused on one of m^6^A-indepdendent targets of rhein, REEP3. We initially examined *Reep3* expression levels in cells cultured in the presence of rhein. At the MCE stage, the expression of *Reep3* increased gradually in the control cells (Figure 5A). Upon rhein treatment, its expression levels substantially reduced in a dose-dependent manner (Figures 5A,B).

To address the role of *Reep3* in regulating adipogenesis, we generated 3T3-L1 cells depleting *Reep3*. As determined by the cell proliferation assay, knockdown of *Reep3* obviously slowed down the cell proliferation rate (Supplementary Figure 7 and Figure 5C). As shown in adipogenic formation assay, ~60–70% control cells were able to differentiate into adipocytes, as determined by ORO staining (Figure 5D). Remarkably, depleting of *Reep3* resulted in severe adipogenesis defects with dramatic reduction of lipid droplets accumulation (Figure 5D). Consistent with the phenotype, knockdown of *Reep3* significantly downregulated the adipogenesis marker genes *Ppar*γ, *Fabp4*, and *Adipoq* (Figures 5E,F). Altogether, these data indicate that REEP3 plays an essential role in driving adipogenic differentiation.

## Discussion

Adipogenesis is defined as the differentiation process from preadipocytes to adipocytes, which undergoes growth arrest of the preadipocytes, accumulation of lipid droplets, and generation of mature adipocytes with specific morphological and biochemical properties (42, 43). This process is accompanied by dramatic transcriptional activation of adipocyte genes including adipocyte FABP and lipid-metabolizing enzymes. MCE after growth arrest occurring within 48 h of adipogenic stimulation is considered as a vital period to support cell growth and induce adipogenic gene expression (8, 44, 45). Rhein is a naturally occurring anthraquinone with a variety of potential pharmacological uses (46), while its functional role in adipogenic differentiation is still largely unknown. We initially characterized the phenotypic effect of rhein on adipogenesis. Intriguingly, treatment of rhein at MCE rather than other stages attenuates 3T3-L1 differentiation with dramatically reduced accumulation of lipid droplets and expressions of adipocyte genes (Figures 1A–F). Rhein predominantly targets the genes highly expressed at the mitotic stage. Supporting this notion, rhein-downregulated genes are associated with the regulation of cell cycle and cell division by using transcriptomic analysis (Figures 2A,F). Moreover, based on the experimental data of cell cycle synchronization, we found that rhein treatment significantly retarded mitotic progression with delayed expressions of mitotic marker proteins, cyclin A and cyclin B1 (Figures 3A–D). Different from its action on adipogenic formation, rhein has been found to inhibit the growth and induce the apoptosis of various cancer cells through interfering ERK1/2, JNK/Jun/caspase, or other pathways (47–52). However, the direct target proteins of rhein in the cell cycle or apoptosis need to be further investigated.

Rhein has been identified to directly bind with FTO and suppresses its demethylase activity (19). We, therefore, compared the transcriptomic response of 3T3-L1 differentiation to rhein treatment and *Fto* depletion in parallel. FTO knockdown or rhein supply suppresses adipogenic differentiation to a similar extent. Surprisingly, only a small subset of genes is commonly downregulated in rhein- and sh*Fto*-treated cells (Figures 2A,B), suggesting that rhein mainly influences adipogenesis independent of regulating FTO activity or functionality. As rhein has also been reported to potentially interact with other AlkB family proteins (12), it will be very interesting to explore whether rhein could target other AlkB proteins to regulate adipogenic formation.

*N*^6^-methyladenosine emerges as an internal modification located on mRNA and non-coding RNAs. Through mediating mRNA stability, translation, and splicing, m^6^A plays crucial roles in the regulation of a broad spectrum of physiological processes including preadipocytes differentiation (53, 54). Knockdown of primary m^6^A demethylase FTO downregulates Janus kinase 2 (*Jak2*) expression in an m^6^A-dependent manner, resulting in suppression of CCAAT/enhancer-binding protein β (*C/ebp*β) expression and adipocyte differentiation (55). Moreover, FTO might exhibit the adipogenesis-promoting effects *via* m^6^A-dependent stability regulation of crucial autophagy factor *Atg5* and *Atg7* (29). Conversely, deficiency of core m^6^A methyltransferase appears to facilitate the progression of adipogenic formation (26). Consistent with the critical function of m^6^A in the adipogenesis process, we found that m^6^A peaks underwent dynamic changes at the different stages of differentiation with a specific increase at 5′ UTR (Figure 4A). The exact functional roles of the boosted 5′ UTR methylation need to be further studied.

We further assume that rhein modulates RNA methylation and adipogenic differentiation potential in an independent manner. There are at least two pieces of evidence supporting this hypothesis. First, the transcripts with upregulated m^6^A levels upon the FTO inhibitor showed similar abundance in the presence and absence of rhein treatment (Figures 4D–F). Second, in the group of 320 rhein-downregulated genes, only a small subset of transcripts bears m^6^A peaks. Furthermore, m^6^A-containing mRNAs were much less sensitive to rhein compared to non-m^6^A transcripts (Figures 4G,H). Perhaps the most exciting finding is the identification of a novel regulator in adipogenic differentiation. REEP3, an endoplasmic reticulum (ER) protein, has been reported to facilitate the correct progression of mitosis, proper nuclear envelope architecture, and maintenance of tubular ER morphology (56, 57). We found that REEP3 was required for driving the expression of adipogenic genes and adipogenic formation (Figure 5). It is very possible that REEP3 guides the proper adipogenic differentiation by targeting MCE progression.

## Conclusion

This study systematically showed the transcriptomic and epitranscriptomic response of the adipocyte differentiation to the phytochemical rhein. It dramatically suppresses adipogenesis at a specific stage in a dose-dependent manner. Additionally, rhein modulates m^6^A methylome rearrangement and adipogenic differentiation in a separate manner. Further, we have identified a novel ER-associated regulator REEP3, which promotes adipogenesis in a m^6^A-independent manner and provides a potential therapeutic target for obesity research.

## Data Availability Statement

The datasets presented in this study can be found in online repositories. The names of the repository/repositories and accession number(s) can be found in the article/Supplementary Material.

## Author Contributions

X-ML and JuZ conceived the project and wrote the manuscript. LH and XZ performed most of the experiments. JZha and XM conducted the high-throughput sequencing data analysis. QL and JG conducted the FTO knockdown-related experiments. JiZ helped with cell culture and data collection. All authors discussed the results and edited the manuscript.

## Funding

This work was supported by grants from the National Natural Science Foundation of China (81974527 and 32070868) and the Natural Science Foundation of Jiangsu Province, China (BK20190553 and BK20211219).

## Conflict of Interest

The authors declare that the research was conducted in the absence of any commercial or financial relationships that could be construed as a potential conflict of interest.

## Publisher's Note

All claims expressed in this article are solely those of the authors and do not necessarily represent those of their affiliated organizations, or those of the publisher, the editors and the reviewers. Any product that may be evaluated in this article, or claim that may be made by its manufacturer, is not guaranteed or endorsed by the publisher.
