# Supplementary material for: The Phytochemical Rhein Mediates M6A-Independent Suppression of Adipocyte Differentiation
Source: Front Nutr. 2021 Nov 1;8:756803. doi: 10.3389/fnut.2021.756803 (PMC8592053; doi:10.3389/fnut.2021.756803)
Supplement: Supplementary file 1 [file Data_Sheet_1.PDF]

The Phytochemical Rhein Mediates m^{6}A-Independent Suppression of Adipocyte Differentiation

Linyuan Huang[1†], Jun Zhang[1†], Xinyun Zhu[1†], Xue Mi[1], Qiujie Li[1], Jing Gao[1], Jianheng Zhou[1], Jun Zhou[1,2*] and Xiao-Min Liu[1*]

[1]School of Life Science and Technology, China Pharmaceutical University, Nanjing, Jiangsu, 210009, China.

[2]State Key Laboratory of Natural Medicines, China Pharmaceutical University, Nanjing, Jiangsu, 210009, China.

[*] Corresponding authors.

E-mail addresses: liuxm642@cpu.edu.cn (Xiao-Min Liu), jz572@cpu.edu.cn (Jun Zhou)

[†]These authors contributed equally.

Running title: Rhein suppresses adipocyte differentiation

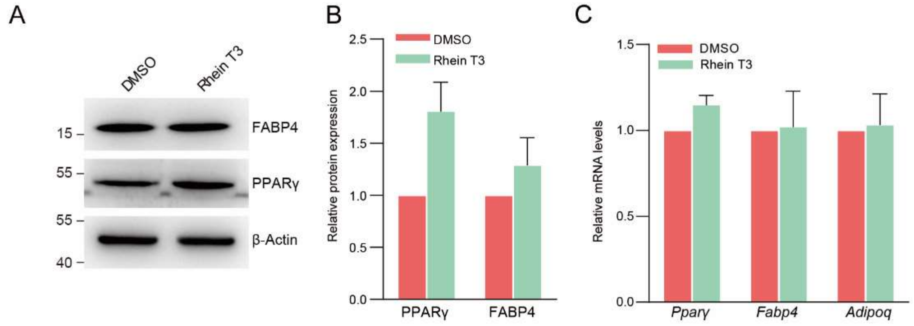

Fig. S1: Long-term rhein treatment shows minor impacts on adipocyte differentiation. A Western blotting of adipogenesis markers. Cells were treated with DMSO or rhein through the MCE and the differentiation stages. Final-differentiated cells were collected for analysis. **B** Quantification of protein levels shown in western blotting. Error bars, mean ± SEM; n = 3 biological replicates. **C** Relative mRNA levels of adipogenesis markers under DMSO or rhein treatment. Error bars, mean ± SEM; n = 4 biological replicates.

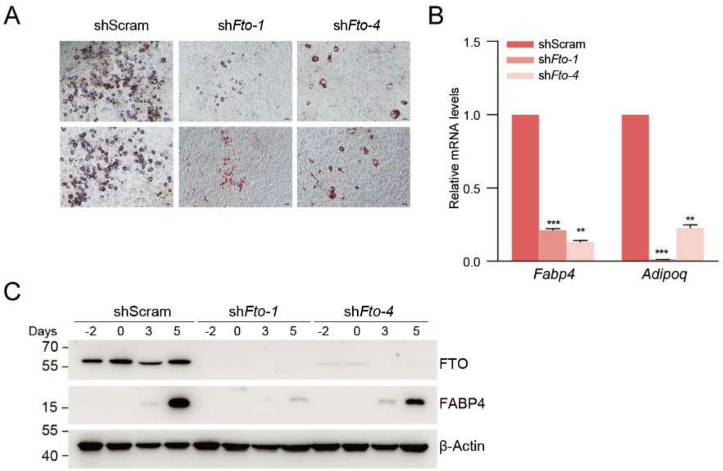

Fig. S2: *Fto* knockdown suppresses adipogenesis. A The adipogenic phenotypes of *Fto* knockdown and Scramble cells. Lipid accumulation was assessed using Oil Red O staining; Scale bars, 5μm. **B** Relative mRNA levels of adipogenesis markers in shScram and sh*Fto* cells. Error bars, mean ± SEM; n = 4 biological replicates; **p < 0.01, ***p < 0.001. **C** Western blotting of adipogenesis markers. shScram and sh*Fto* cells were induced to generate adipocytes by standard MDI medium, and final differentiated cells were collected for analysis.

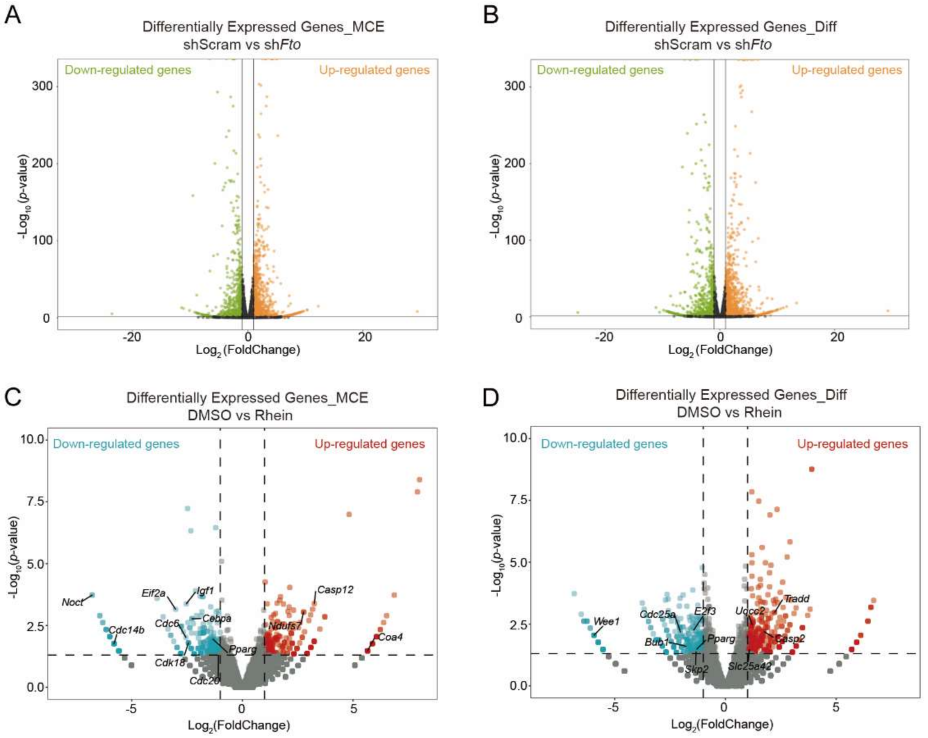

Fig. S3: *Fto* knockdown and rhein influence transcriptome. A and B Volcano plot showing differentially expressed genes (DEGs) in shScram or sh*Fto*cells collected at the MCE stage or the differentiation stage, respectively. **C and D** Volcano plot showing DEGs in DMSO or rhein-treated cells collected at the MCE stage or the differentiation stage, respectively. For both A and B, orange dots represent significantly up-regulated DEGs, and green dots represent significantly down-regulated genes. For both C and D, red dots represent significantly up-regulated DEGs, and blue dots represent significantly down-regulated genes.

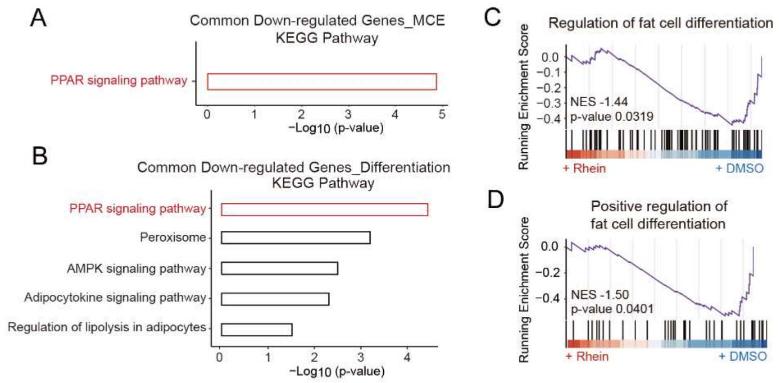

Fig. S4: Rhein altered expression levels of mitotic-related genes. A and B KEGG pathway analysis of common down-regulated genes in rhein-treated and *Fto* knockdown cells collected at the MCE stage and the differentiation stage, respectively. **C and D** Gene-set enrichment analysis plots for rhein-regulated genes, as determined by RNA-seq.

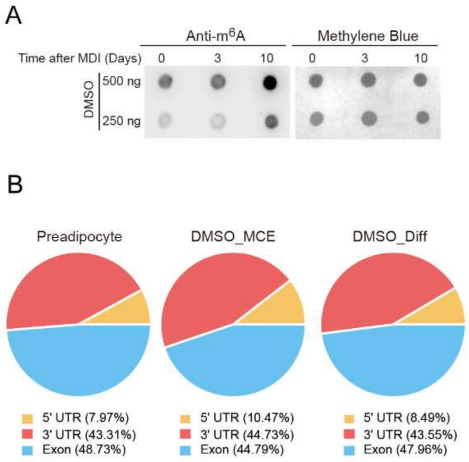

Fig. S5: m^{6}A methylome altered during adipogenesis. A m^{6}A dot blotting represents m^{6}A levels across adipogenesis. **B** Pie chart presenting fractions of m^{6}A peaks in different transcript segments.

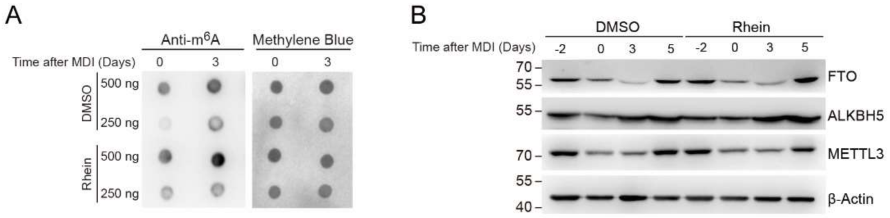

Fig. S6: Impacts of rhein on m^{6}A installation at distinct stages of adipogenesis. A m^{6}A dot blotting represents m^{6}A levels under DMSO or rhein treatment on day 0 and day 3. **B** Western blotting ofm^{6}A vital regulators. Cells were treated with DMSO or rhein and collected at indicated time points for analysis.

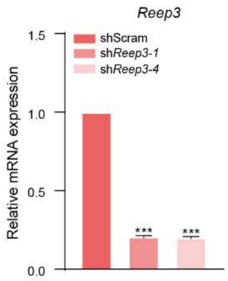

Fig. S7: Knockdown efficiency of sh*Reep3*. Relative mRNA levels of *Reep3* in shScram and sh*Reep3* cells. Error bars, mean ± SEM; n = 6 biological replicates; **p < 0.01, ***p < 0.001.

Table S1. Sequences of qPCR primers

Gene	Species	Sequences (5'-3')
Actb-Fwd	*Mus musculus*	TTACTGCTCTGGCTCCTAGC
Actb-Rev	*Mus musculus*	CCTGCTTGCTGATCCACATC
Pparγ-Fwd	*Mus musculus*	TGTTATGGGTGAAACTCTGGG
Pparγ-Rev	*Mus musculus*	AGAGCTGATTCCGAAGTTGG
Fabp4-Fwd	*Mus musculus*	GACAGGAAGGTGAAGAGCATC
Fabp4-Rev	*Mus musculus*	GTCACGCCTTTCATAACACATTC
Adipoq-Fwd	*Mus musculus*	TCAACGACTCTACATTTACTGGC
Adipoq-Rev	*Mus musculus*	TCGACTGTTCCATGATTCTCC
CyclinA-Fwd	*Mus musculus*	GTCCTTGCTTTTGACTTGGC
CyclinA-Rev	*Mus musculus*	ACGGGTCAGCATCTATCAAAC
CyclinB1-Fwd	*Mus musculus*	GACTCCCTGCTTCCTGTTATG
CyclinB1-Rev	*Mus musculus*	GCTGCATACTTGTTCTTGACAG
Reep3-Fwd	*Mus musculus*	AAGATGGAAGTGAGCAGACAG
Reep3-Rev	*Mus musculus*	TTTAGTGAGCCATACCGCAC
